# Evaluating the Homogeneity of Surface Features Induced by Impact-Based Surface Treatments

**DOI:** 10.3390/ma14133476

**Published:** 2021-06-22

**Authors:** Asghar Heydari Astaraee, Sara Bagherifard, Stefano Monti, Mario Guagliano

**Affiliations:** Department of Mechanical Engineering, Politecnico di Milano, 20156 Milan, Italy; asghar.heydariastaraee@polimi.it (A.H.A.); stefano.monti@polimi.it (S.M.); mario.guagliano@polimi.it (M.G.)

**Keywords:** impact-based surface treatments, shot peening, surface roughness, surface morphology

## Abstract

Impact surface treatments are well-known for their efficiency in enhancing the mechanical properties of metallic materials, especially under cyclic loadings. These processes, which encompass a wide range of surface treatments based on repetitive impacts of tools of various types, induce surface plastic deformation, compressive residual stresses, and grain refinement alter the surface roughness as a side effect. Thus, it is essential to have suitable indexes to quantify the surface features caused by the typically random nature of these treatments. Herein, we evaluated the rationality of using standard roughness parameters for describing the morphological characteristics of surfaces treated by shot peening as a representative and widely used treatment of the category. A detailed numerical model of the peening process was developed. The output data were elaborated to extract the surface roughness parameters following the standard procedures. The results revealed the validity of the surface roughness parameters to describe the topography of material treated with adequate surface coverage, also highlighting the necessity to use a set of parameters rather than the common practice of relying on single parameters. Not considering a comprehensive set of amplitude and spacing parameters can result in significant, inconsistent, and misleading results while comparing the performance of surfaces.

## 1. Introduction

Surface features defined in terms of roughness or morphology are of utmost importance in directing materials’ interaction with their surrounding environment and their performance under applied static and, particularly, fatigue loads. Furthermore, geometrical accuracy and aesthetic features are highly affected by surface morphological features. Thus, several standards have been developed to put forward a universal approach for describing the geometrical specification of surfaces using profile or areal parameters. Some commonly used examples are ISO 25178 [1], ISO 4287 [2], ISO 4288 [3], DIN 4762 [4], and JIS B 0601 [5].

Despite the exhaustive list of surface texture parameters suggested by these standards, the majority of the studies, particularly in the field of impact-based surface treatments, refer to the single parameter of the arithmetical mean deviation (*Ra*/*Sa*) [6,7,8] or root mean square deviation (*Rq*/*Sq*) [9] of the assessed profile/surface as the only index to describe surface roughness. These most commonly used parameters are occasionally accompanied by *Rt*/*St* or *Rz*/*Sz* [10,11,12] that are all local parameters directly related to the distance between the highest peaks and the lowest valleys in the measured profile/area. More recent investigations revealed that this particularly limited set of parameters can lead to misleading conclusions when comparing dissimilar surfaces [13,14]. Surfaces with similar *Ra* could have a distinctly different spatial distribution of the peaks and valleys. Likewise, surfaces can have the same peak to valley distance while exhibiting distinctly different organization of surface features. It is also noted that the reproducibility of some roughness parameters is not guaranteed [15], which could add to the uncertainty. Failing to notice these delicate aspects has resulted in significant inconsistent and contradictory results while comparing the performance of different surfaces in various fields of applications. As an example, it is widely accepted that to account for the effect of surface roughness on the fatigue strength, a “surface coefficient” that is a function of *Rt* is used; however, samples with a certain *Rt* machined or treated in a different way, will have quite different behavior with respect of the one that can be determined when the corresponding surface coefficient is applied [16].

These issues can become even more challenging when dealing with surfaces characterized by random or disordered geometrical features. Surfaces treated by impact based treatments, including shot peening [17], sand blasting [18], ultrasonic shot peening (USP) [19], surface mechanical attrition treatment (SMAT) [20], and similar procedures are typical examples showing a high density of randomly positioned overlapping peaks and valleys. Shot peening is a mechanical surface treatment, which has been rigorously studied, researched, and improved in the last couple of decades for a wide range of applications in different fields ranging from automotive to aeronautical and bio-medical sectors. It is mainly used for enhancing fatigue performance of metallic parts [21,22,23,24,25,26,27], surface nanocrystallization [28,29,30], bio-functionalization [31,32,33], modulating wear and corrosion resistance [34,35,36], and surface texturing [37,38,39]. All the properties mentioned above are highly interrelated with the surface features of the shot-peened material. Due to the random sequence and the random impact positioning of small shots that are accelerated by compressed air or by a centrifugal wheel during shot peening, the treated surfaces are typically characterized by irregular surface features. This irregularity is further accentuated because of imperfect surface quality and insufficient sphericity of the impacting media. Likewise, in the case of shot peening, most studies have based their comparison on the frequently used parameters of *Ra* and *Rz*, even if surfaces with similar values of this latter can exhibit quite different behavior due to a different overall surface features arrangement and a different texture. Numerous studies [40,41,42,43,44,45] have been performed on the numerical investigation of surface roughness prediction induced by peening, taking for granted the validity of the standard roughness parameters.

Bearing the above discussion in mind, most functions of the shot-peened surfaces, including fatigue performance, fretting, wear and corrosion resistance, wettability, and interaction with the biological environment, etc., are highly entangled with the surface roughness features. This study aims to evaluate the applicability of standard roughness parameters for characterizing shot-peened surfaces from low to full surface coverage levels. The two important aspects under study are the repeatability and the comprehensiveness of the roughness data. The former regards the intrinsic randomness of the multiple shot impacts as the main source of the surface topography evolution. At the same time, the latter evaluates that how accurate the description of the standard surface topography parameters would be in the case of using just a few of the most commonly used parameters (i.e., *Ra* or *Rz*) and neglecting the others. We believe that this area has been poorly studied, especially for the specific case of peening treatments. Thus, this study sheds light on the validity of standard roughness parameters for peened surfaces and suggests methods that can enhance the reliability of surface roughness quantification based on such roughness parameters.

To this aim, a series of numerical models of the shot peening treatment have been developed considering pure iron as a representative material for which we have experimental data for comparison. The developed model was validated and run several times, considering varying arrangements for the impacting shots. A MATLAB (The MathWorks, version was 9.9, R2020b release)code was developed based on the instructions and the description of parameters provided by standards to extract an extensive list of roughness parameters from the simulation outputs and provide a comprehensive description of the disordered surface features. The evolution of these parameters was studied as a function of the exposure time and compared with the roughness parameters experimentally measured on samples treated with the same peening process used in the numerical simulations. Comparison of the numerical surface roughness data produced by varied positioning and sequence of the impacts with the experimental data indicated the importance of the individual and combined set of roughness parameters in providing meaningful and reliable surface roughness indexes for irregular peened surfaces. This could be used for quantitatively assessing the final surface state and its effect on the performance in terms of fatigue strength and also in other possible fields of applications.

## 2. Materials and Methods

### 2.1. Experimental Tests

Cubic samples (30 × 30 × 5 mm^3^) of Armco pure iron (99.89%) were air blast shot peened using an Almen intensity of 6 N (0.001 in.) and surface coverage of 100%. AZB100 Zirconia shots with a nominal diameter of 100 µm were used as the peening media.

An Alicona Infinite Focus optical microscope (Bruker Alicona, Graz, Austria) was used to scan an area of 10.2 × 5.6 mm^2^ on the surface of the samples with a 5× lens with lateral and vertical resolution ranges of 3.5–23.5 µm and 0.410–23.0 µm, respectively. The rather large area of the scan was intended to have a proper averaged evaluation of the surface roughness using smaller sub-surfaces for averaging. Standard profile and areal roughness parameters were extracted from the scanned file to serve as reference data for comparison with the roughness parameters obtained from the numerical models.

### 2.2. Numerical Modeling and Code Development for Data Extraction

**Figure 1:**
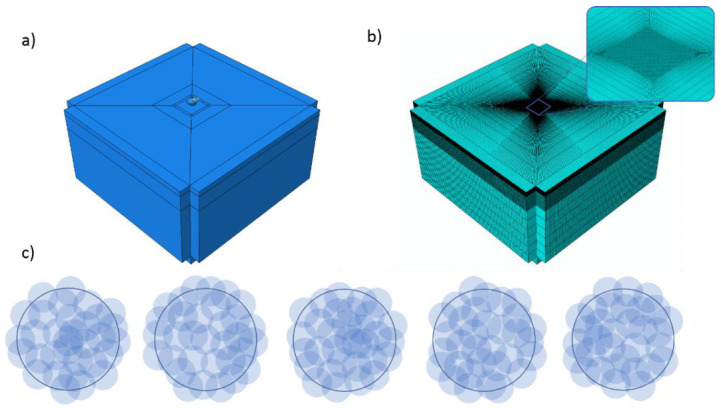
(**a**) Geometry (**b**) and mesh details of the numerical model; (**c**) the comparison between the arrangements of the shots in the five FE analyses.

Finite element (FE) simulations were performed using ABAQUS Explicit 2019. The model consisted of a body as the target surface and a group of shots impacting on its surface in a pre-defined random sequence. To avoid models as large as the sample, it is customary to use a 3D confined cell as a representative volume, hereafter called the target. The details of the model are depicted in Figure 1. The dimensions of the target were selected to be 1.5 × 1.5 × 1.0 mm^3^ based on the authors’ previous investigations [46]. An area equal to 0.2 × 0.2 mm^2^ in the center of the top surface of the target was partitioned to enable the generation of a finer mesh for more accurate results. This contains the central impact area on which shot impingement occurred. The size of the impact area was chosen regarding a trade-off between the accuracy of the results and the computational cost of the analysis concerning the total element number. The target was meshed using C3D8R elements (3D eight-node hexahedral with reduced integration). The outer faces of the target except the top surface were meshed by half infinite elements to reproduce a large surrounding body and to avoid the reflection of elastic shear waves [47]. The mesh size in the impact area was refined to 1/20th of the diameter of the dimple induced on the surface by a single shot impact. The ceramic media were modeled as mesh-less rigid body spheres to minimize the computational cost of the analysis. The assumption of a rigid body is valid since the hard and brittle nature of the ceramic media leads to almost no deformation in the shot upon impact with the much softer iron specimen. The diameter of the shot (d) was considered as the nominal diameter of the ceramic media equal to 0.10 mm.

The material parameters of the model are provided in Table 1. Based on the density (ρ), the mass (*m*) and moment of inertia (*I*) were calculated for the sphere and assigned to the shot. The plastic flow of the Armco iron^®^ was modeled using the rate-dependent Johnson-Cook model. The relevant parameters of the model [48] along with the density and elastic material properties are shown in Table 1.

The interaction between the target and the shots was defined using a tangential behavior with a friction coefficient (μ) equal to 0.2 and a general contact algorithm. Owing to very large deformations induced by shot peening at higher coverage levels, the built-in routine of the arbitrary Eulerian-Lagrangian meshing technique was applied. The velocity was assigned to the impacting shots as an initial condition. Thermal effects were considered to be negligible. The step time was set such that the residual stress of a single impact along the central path under the indent was stabilized. The step time was used to set the distance between successive shots in the multiple impact model.

The multiple impact shot peening model was developed using a developed Python subroutine. The positioning of the shots with a prescribed initial velocity was accomplished by the subroutine. A random function was utilized to produce the randomized coordinates of the shots within the impact area. The subroutine was capable of defining the optimal position of each shot required to achieve the desired coverage level. Once the dimple diameter of a single impact and the dimension of the total impact area were determined, the subroutine selected 30 points located randomly within the impact area at each loop of the coverage calculation step. Starting from the first randomly positioned shot, the subroutine calculated the surface coverage by considering all the 30 points as a candidate for the position of the next impact. The final choice for the shot position of each impact was such that it resulted in the highest surface coverage among the 30 points. Then, the procedure continued with the consecutive impacts until the desired surface coverage level was reached within the impact area.

A dedicated MATLAB code was developed for data analysis and post-processing of the simulation results. The code extracted the original and deformed coordinates of all points on the target surface of the model after multiple impacts. It then meticulously followed the description provided by the standards of geometrical specification of surfaces for calculating the roughness parameters from the deformed profile, considering it as a waveform signal. The developed code used an interpolation function to reproduce the surface features with high accuracy. This signal is composed of two main contributions of waviness and roughness. The standard requires the waviness contribution to be removed before estimating the roughness parameters. Thus, the low-frequency components were filtered from the primary profile; this was performed through a convolution in the space domain between the primary profile and the Gaussian filter. Considering the surface obtained from the numerical simulation as a series of roughness profiles, for each row and column of the extracted surface (a total of 40,000 profiles), a filtering process was applied using a Gaussian filter to separate the low-frequency components. The resultant data included the higher frequency components solely and were used to estimate the roughness parameters. Apart from the areal parameters, which evaluate the impacted target surface as a whole, profile parameters were also calculated for each row and each column within the target area.

The average heights of the profile/area were calculated for individual profiles and the whole target surface of the model. These parameters served as a new reference height that was subtracted from the Z-coordinate of all nodes on the corresponding profile/area, as required by the standard, before calculating the roughness parameters. This correction also eliminated the influence of any residual contribution of the waviness on the roughness parameters. The roughness parameters calculated on the individual profiles were then used to estimate the average values and the resulting standard deviations, for each profile roughness parameter, as described later in the results section.

### 2.3. Description of Select Roughness Parameters

The standard corresponding to surface texture and morphology specifications, ISO 4287, provides an exhaustive list of parameters that describe the geometrical features of the surface. Herein, we have selected a broad list of these standard surface roughness parameters that we assume to be more relevant and expressive for peened surfaces and their prospective mechanical performance. In particular, these parameters deal with amplitude (vertical distribution of surface deviations), spacing (horizontal distribution of surface deviations), and hybrid aspects in a way that provides a holistic description of the surface features. These parameters can be mostly outlined both as profile and areal factors. Profile parameters are defined based on a sampling length, the length used for identifying the irregularities of the profile under investigation, as described in ISO 4287. Profile parameters are normally calculated on multiple sampling lengths and then averaged according to ISO 4288. Areal parameters, on the other hand, are calculated on the whole scanned/simulated target area.

Table 2 and Table 3 provide a brief description for each group of profile and areal parameters included in this study reporting. The selected amplitude parameters in this work include (i) arithmetic mean height (*Ra*) that is the average absolute deviation of profile irregularities from the mean line over the profile length; (ii) root mean square (*Rq*, *RMS*) that is the standard deviation of the surface height distribution and is more sensitive to large deviations from the mean line compared to *Ra*; (iii) ten-point height (*Rz*) that is the height difference between the average of the five highest peaks and the five lowest valleys along the profile; (iv) skewness (*Rsk*) that is used to distinguish between two profiles having the same *Rv* values but with different shapes and different height distributions. *Rsk* measures the symmetry of the profile about the mean line; (v) kurtosis (*Rku*), which describes the peakedness of the profile. A surface with a narrow height distribution has a kurtosis value greater than 3; while a surface that has a well spread-out height distribution has a kurtosis value of less than 3. *Rku* can be used to differentiate between surfaces with the same *Ra* but dissimilar shapes.

We also included a spacing parameter, which is the mean spacing of adjacent local peaks (*S*). It is defined as the average spacing of adjacent local peaks of the profile measured along the assessment length. The local peak is defined as the highest part of the profile measured between two adjacent minima and is only measured if the vertical distance between the adjacent peaks is greater than or equal to 10% of the absolute peak to valley (*Rt*) of the profile.

Regarding the areal parameters, the list, including amplitude, spacing, and hybrid parameters, consists of (i) arithmetic mean height (*Sa*) that is the arithmetic mean of the absolute value of the height within the sampling area; (ii) root mean square height (*Sq*) that is the root mean square value of the height distribution within the sampling area; (iii) ten-point height (*Sz*) that is the average difference of the five highest points and five deepest valleys over the sampling area; (iv) areal skewness (*Ssk*), which is described as the ratio of the mean of the height values cubed and the cube of *Sq* within a sampling area; (v) areal kurtosis (*Sku*) that is considered as the measure of the sharpness of the surface height distribution calculated as the ratio of the mean of the fourth power of the height values and the fourth power of *Sq* within the sampling area, and (vii) developed interfacial area ratio (*Sdr*) that is the percentage of the definition area’s extended surface contributed by the texture as compared to the planar area.

## 3. Results

The proposed numerical model was validated by comparing the induced residual stresses from the numerical simulation and experiment in a previous study [33]. The FE analysis was run with five different impact sequences and arrangements for 32 shots needed to guarantee 100% coverage using the random function imbedded in the subroutine developed for model creation; these analyses could provide insight into the state of roughness at different locations on the surface of the treated material in practice. The MATLAB code was run on the deformed surface obtained from each of the five FE analyses to extract the deformed shape, calculate the select standard roughness parameters, and compare them with the experimental data. The experimental data presented here refer to the parameters estimated by the developed MATLAB code on the whole area scanned by the microscopical analysis and on the multiple horizontal and vertical profiles within it. Considering the different sizes of the areas scanned in the experiment and those of the numerical models (that were scaled down due to computational costs), the cut-off wavelengths of 0.25 mm and 0.08 mm were considered for the corresponding analysis.

**Figure 2:**
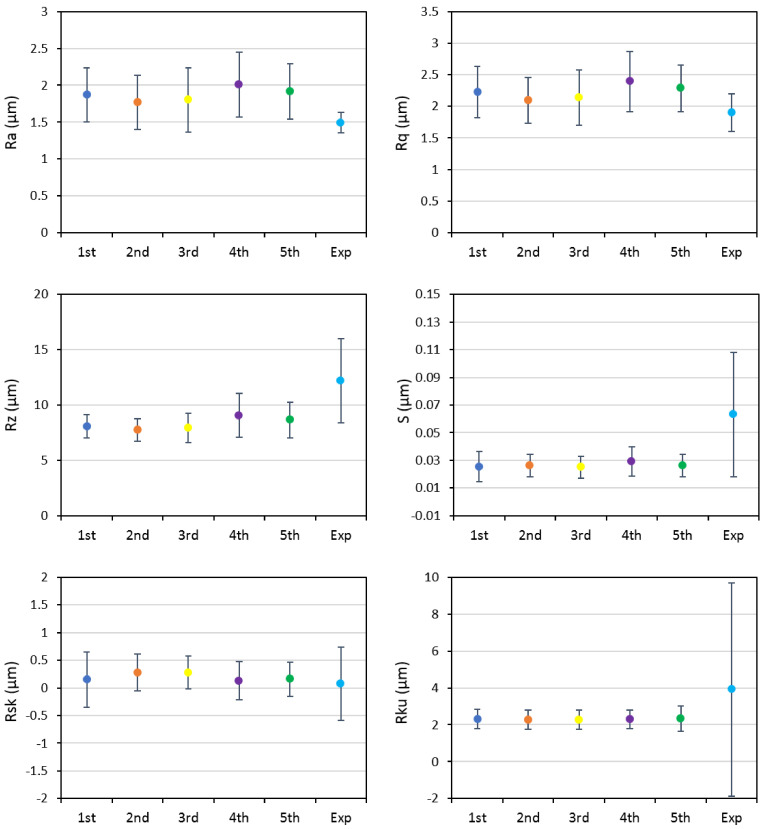
Comparison of profile parameters obtained from five different simulations and the experimental results for full coverage.

**Figure 3:**
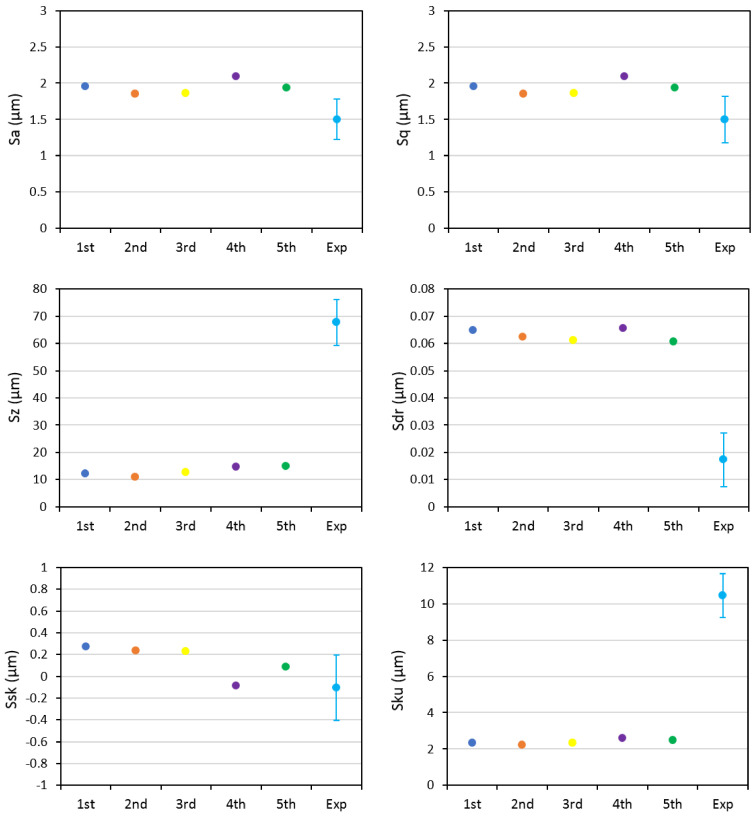
Comparison of areal parameters obtained from five different simulations and the experimental results for full coverage.

The graphs in Figure 2 and Figure 3 compare multiple profiles and areal roughness parameters, respectively, for the numerical analysis and experimental tests. The profile parameters included amplitude parameters of *Ra*, *Rq*, *Rz*, *Rku*, and *Rsk*. Mean spacing of adjacent local peaks (*S*), as a spacing profile parameter, was also included in Figure 2. The obtained results indicated a very good match between the five numerical simulations; for most of the analyzed parameters, the numerical results showed a good agreement with the experimental data, as well. The more local parameters, including *Rz* and *S*, showed a higher scatter in the case of the experimental data. Experimentally calculated *Rku* showed the highest scatter among the analyzed profile parameters.

Figure 3 represents the comparison between areal parameters obtained from different runs of the numerical simulation and the experimental data. In these analyses, all parameters were kept constant apart from the order and sequence of the multiple impacts; thus, to represent the randomness of the process itself. Similar to the profile data analysis, the areal parameters also indicated a slight variation between the results of different numerical runs; this difference highlighted the effect of shot arrangement and sequence on surface morphology; however, while the differences observed in the case of profile parameters between the experimental and numerical results was more within the range of the data scatter, the areal data represented a more significant deviation compared to the experimental data. This difference was more remarkable in the case of *Sz*, *Sdr*, and *Sku* parameters.

**Figure 4:**
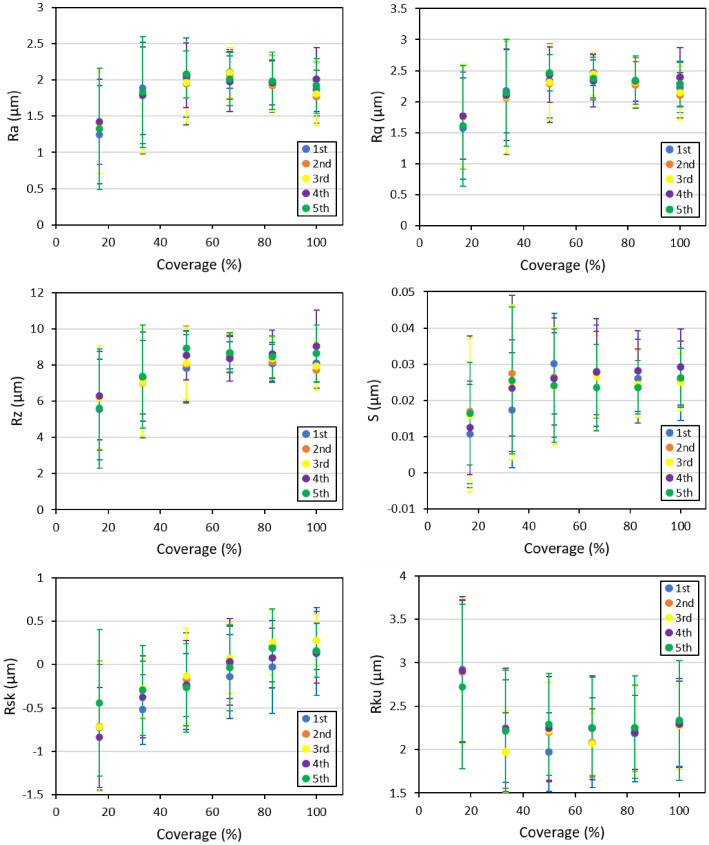
Numerical results on the evolution of select profile parameters as a function of surface coverage.

**Figure 5:**
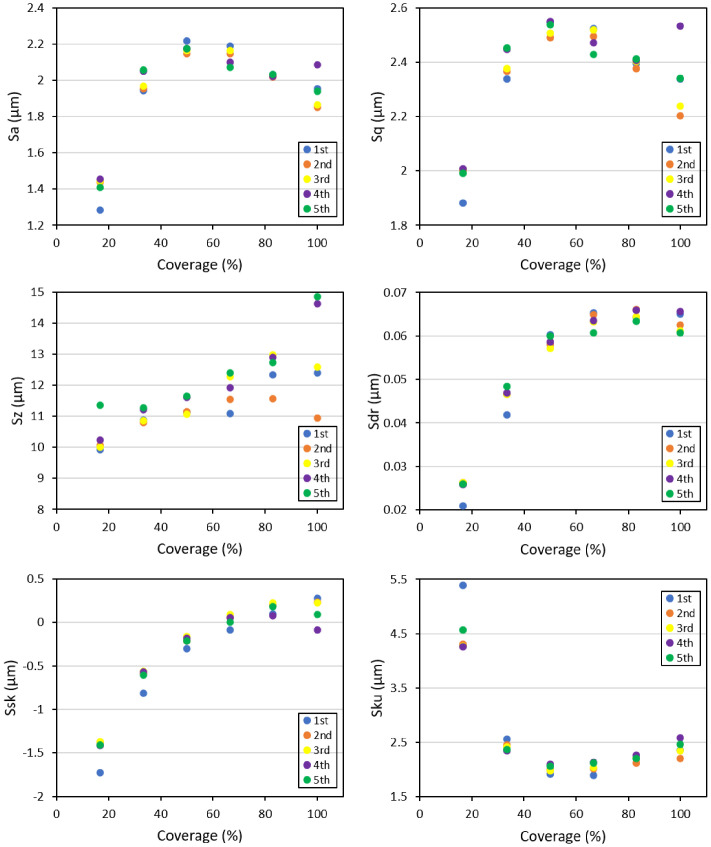
Evolution of select areal parameters as a function of surface coverage in the numerical simulations.

Figure 4 and Figure 5 show, respectively, the evolution of select profile and areal roughness parameters as a function of coverage in the numerical simulations. The presented values at each coverage were the average of measurements performed on all horizontal and vertical lines within the scanned area. The profile parameters of *Ra*, *Rq*, and *Rz* showed an initial increase of up to around 50% coverage followed by a plateau. *Rsk* also showed a similar trend, despite representing a slower rate of saturation with respect to the aforementioned amplitude parameters. Rku, on the other hand, represented a different trend of initial decrease and then stabilized after 40–50% surface coverage. The mean spacing between the neighboring peaks became stabilized at quite low surface coverages, and its scatter reduced by increasing the surface coverage.

Regarding the areal parameters, *Sa* and *Sq*, the trend was quite similar to *Ra*, *Rq*; however, at higher surface coverages, the stabilized values tended to decrease. *Sz* behavior was quite different from the rest of the amplitude parameters representing a continuous increase characterized with a higher scatter between different simulations compared to all the other areal parameters. *Sdr* showed an increasing and then stabilized trend at higher exposure times. *Ssk* and *Sku* confirmed the trends represented by *Rsk* and *Rku*, respectively.

## 4. Discussion

**Figure 6:**
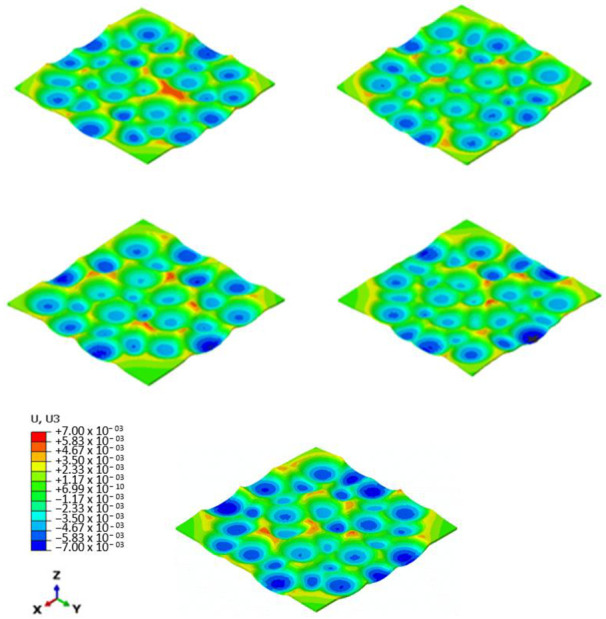
Qualitative comparison of the top surface morphology of the numerical simulations performed using the same process parameters but with different shot arrangements and impact sequences.

The data obtained from five different runs of the validated FE model were compared. Owing to the randomness of the impacts in the shot peening process, the surface morphologies obtained from different analyses were not identical despite the fact that they were treated with the same process parameters (peening medium, intensity, and surface coverage). Figure 6 represents the surface morphologies obtained from these different impact sequences and positionings. Herein, we evaluated to what extent these qualitative differences could be reflected in standard roughness parameters.

The results of the simulations confirmed that shot peening generally increased the surface roughness of the treated surface, starting from a smooth morphology. The evolution of different profile and areal parameters varied as a function of surface coverage, although a general theme could be observed for the majority of the analyzed parameters.

The profile parameters, including *Ra*, *Rq*, and *Rz*, showed an initial increase and became saturated, representing a plateau after around 50% surface coverage for the simulated peening parameters. The initial rapid increase in most profile and areal parameters could be due to the formation of individual dimples that were mainly separated from each other at lower exposure times. In the beginning, most of the target surface was uncovered, and thus, individual impacts and the resulting craters had a notable effect in inducing high local peaks and deep valleys, leading to sharp jumps in roughness parameters. With the increase in coverage, the distances between adjacent dimples reduced, resulting in more homogeneous surface morphology and thus a decreased slope of roughness profile as a function of exposure time. The stabilization of the surface roughness parameters at higher exposure times could be attributed to the fact that at relatively higher surface coverages where a more normal distribution was obtained for the location of impacted areas, the rate of peak and valley formation became comparable with the rate of peak height reduction [49].

Rsk showed a slower stabilization trend starting from values close to −1 and reaching values under 0.5 at higher coverages; this indicated that as the surface coverage increased, the distribution of peaks and valleys tended to become more and more symmetric. Rku, on the other hand, represented a dissimilar trend as it shows an initial decrease before becoming stabilized at 40–50% surface coverage. Rku values lower than three for all surface coverages imply a relatively well spread distribution of peaks in the profiles; the decreasing trend as the surface coverage increased suggests that the distribution of peaks became more and more regular until it stabilized at 50% and remained almost constant in terms of average values.

The areal parameters provided a more global description of the surface state. In most cases, they confirmed the trends represented by the corresponding profile parameters. *Sa* and S, showed the swift initial increase up to 50% coverage. They represented stabilized values up to around 70% coverage after which a slight reduction and a sudden divergence could be observed in the values, especially at full coverage. This divergence was also noted for Sz parameter that, contrary to other parameters, kept an increasing trend.

Compared to the corresponding profile parameter (*Rz*), *Sz* seemed to be more affected by local peaks and thus did not stabilize even at higher exposure times, at least up to the full coverage that was the maximum coverage considered in this set of analysis. The high scatter observed for this parameter confirmed its sensibility as the most local parameter in the list. *Sdr*, which is an index of increase in the surface area, showed a swift initial increase as individual dimples were generated on the surface, but as the surface coverage increased and fewer areas remained not impacted, this parameter became stabilized at coverages around 70%. Regarding *Ssk* and *Sku*, both confirmed the same trends represented by the corresponding profile parameters indicating that as the surface coverage became closer to full coverage, the height distribution and the peakedness of the profiles became more and more homogeneous.

Comparing the results of the five numerical simulations indicated that although the sequence and position of subsequent impacting media in the shot peening process followed a random pattern, the target surfaces could be generally characterized by a relatively regular distribution of plastic dimples if sufficiently high surface coverage is considered. This relative stabilization was observed for the majority of the profile and areal parameters to be around 50% coverage. Despite the large scatter at different time points, the numerical results illustrate a good match between the average values obtained for all five analyses. Therefore, the commonly used standard surface roughness parameters seem to be valid to describe the surface topography of shot-peened material.

When compared with the experimental roughness measurements, for most of the averaged profile parameters, including *Ra*, *Rq*, and *Rsk*, and the corresponding areal parameters (*Sa*, *Sq*, and *Ssk*), the numerical data exhibited a very good match with the experimental data. *Rz*, *S*, and especially *Sz*, which are intrinsically local parameters, however, showed a notable difference between the average values of the numerical analysis and the experimental measurements. The inherent randomness of the process paired with the localness of these parameters could somehow justify the noted differences. The significant difference observed in the case of *Sz* parameter could be attributed to the much larger scan area of the experimental tests compared to the smaller target areas considered for the numerical analysis to retain the computational costs.

*Rsk* and *Ssk* showed similar trends for all cases with values around zero, highlighting the approximate symmetry of the surfaces and profiles. *Sku* showed the highest deviation between the numerical and experimental data. While all the numerical analyses were characterized by profile and areal kurtosis values lower than three, indicating a well spread-out height distribution, the experimental data showed much higher values. The divergence of *Sku* could somehow be induced by the significant scatter of the Rku measured experimentally on the surface of the peened sample.

## 5. Conclusions

Since most functions of the shot-peened surfaces, including fatigue performance, fretting, wear and corrosion resistance, wettability, and general interaction with the immediate environment, are highly entangled with the surface roughness, it is important to have reliable and repeatable measures to characterize the surface morphology of peened surfaces. The variation of profile and areal roughness parameters were investigated for multiple random arrangements of subsequent impacts using a detailed numerical model of the shot peening process. The obtained results were compared with those measured on experimentally shot peened samples.

A very good agreement was achieved between the numerical and experimental roughness data at full coverage concerning most of the averaged profile and areal parameters. The differences between the numerically estimated and experimentally measured data in terms of profile parameters were in all cases within the range of the data scatter; however, with no possibility of averaging and thus with no scatter bands available, the areal data represented a more significant deviation compared to the experimental measurements. Nevertheless, based on the trend and the obtained values, these observations validated the proposed numerical model.

The results of the performed analyses indicated how various standard profiles and areal roughness parameters evolve with surface coverage. In most cases, an initial swift increase was followed by a plateau and stabilized evolution at higher exposure times. This initial rapid increase could be attributed to the fact that at the beginning, most of the target surface was uncovered and thus individual impacts and the resulting craters had a notable effect in inducing high local peaks and deep valleys leading to sharp jumps in roughness parameters. The more local parameter, however, continued to stabilize at a much lower rate and, in some cases, maintained the increasing trend up to full coverage.

The qualitative analysis of surface morphologies obtained from numerical simulations run with different sequences and arrangement of impacts exhibited different local distribution of the peaks and valleys. However, these differences were missed when the averaging approach required to calculate the standard roughness parameters was applied. Indeed, the average standard roughness parameters showed a good match between all the analyses, all converging to similar data. This could be an indication that despite local differences, the surfaces obtained from shot peening treatment could exhibit a general homogeneous morphology, if described by the standard surface roughness parameters. However, the apparent differences in the surface morphologies indicate that individual standard roughness parameters may not be able to accurately represent the topological features induced by the randomly positioned impacts of shot peening. This could become an issue for describing some surface functions that depend on specific morphological features. In these cases, with the current resources and if the standard roughness parameters are to be used as a reference, a combination of amplitude, spacing, and hybrid parameters should be used to describe the morphological aspects of shot-peened surfaces, including the form of irregularities and their spatial distribution.

Further studies are needed to define new sets of more indicative roughness parameters and also to completely understand the role of surface morphology on the mechanical properties of shot-peened surfaces and to develop methods for the assessment of shot peening effects that can account for the surface state. This is needed to lead the choice of the peening parameters, considering not only the residual stress distribution but also how shot peening is able to modify the surface morphology.

## Figures and Tables

**Table 1:** Material parameters used in the modeling (data taken from [48]).

**Ceramic Media**	
Density, ρ (g/cm^3^)	5.35
Mass, *m* (g)	2.88 × 10^−6^
Moment of inertia, *I* (g.mm^2^)	2.88 × 10^−9^
**Armco Iron^®^**	
Density, ρ (g/cm^3^)	7.89
Elastic modulus, *E* (GPa)	207
Poisson’s ratio, ϑ	0.29
Elastic limit, *A* (MPa)	175
Hardening constant, *B* (MPa)	380
Hardening exponent, *n*	0.32
Strain rate constant, *C*	0.06
Reference strain rate, $\dot{\varepsilon }$_0_ (s^−1^)	1.0

**Table 2:** Description of the select profile parameters.

Areal Parameter	Mathematical Definition
**Amplitude Parameters**	
Arithmetic mean height	$Ra=\frac{1}{l}\int_{0}^{l}\left|y\left(x\right)\right|dx$
Root mean square roughness	$Rq=\sqrt{\frac{1}{l}\int_{0}^{l}{\left\{y\left(x\right)\right\}}^{2}dx}$
Ten-point height	$Rz=\frac{1}{n}\left(\overset{n}{\underset{i=1}{\sum }}p_{i}-\overset{n}{\underset{i=1}{\sum }}v_{i}\right)$
Skewness	$Rsk=\frac{1}{Rq^{3}}\int_{-\infty }^{\infty }y^{3}p\left(y\right)dy$
Kurtosis	$Rku=\frac{1}{Rq^{4}}\int_{-\infty }^{\infty }y^{4}p\left(y\right)dy$
**Spacing Parameters**	
Mean spacing of adjacent local peaks	$S=\frac{1}{N}\overset{n}{\underset{i=1}{\sum }}S_{i}$

**Table 3:** Description of the selected areal parameters.

Areal Parameter	Mathematical Definition
Arithmetic mean height	$Sa=\frac{1}{A}\iint \left|z\left(x,y\right)\right|dxdy$
Root mean square height	$Sq=\sqrt{\frac{1}{A}\iint \left|z\left(x,y\right)\right|dxdy}$
Maximum peak to valley height	$Sz=Sp+\left|Sv\right|$
Skewness	$Ssk=\frac{1}{Sq^{3}}\frac{1}{A}\iint z^{3}\left(x,y\right)dxdy$
Kurtosis	$Sku=\frac{1}{Sq^{4}}\frac{1}{A}\iint z^{4}\left(x,y\right)dxdy$
Developed interfacial area ratio	$Sdr=\frac{1}{A}\left[\iint_{A}^{\;}\left(\sqrt{\left[1+{\left(\frac{\partial z\left(x,y\right)}{\partial x}\right)}^{2}+{\left(\frac{\partial z\left(x,y\right)}{\partial y}\right)}^{2}\right]}-1\right)dxdy\right]$

## Data Availability

Data is available upon request from authors.
